# Exploring risk pooling in hospitals to reduce demand and lead time uncertainty

**DOI:** 10.1007/s12063-020-00171-y

**Published:** 2020-12-11

**Authors:** Gerald Oeser, Pietro Romano

**Affiliations:** 1 Faculty of Business and Health, Bielefeld University of Applied Sciences, Bielefeld, Germany; 2 Department of Electrical, Management and Mechanical Engineering, University of Udine, Udine, Italy

**Keywords:** Risk pooling, Demand uncertainty, Lead time uncertainty, Logistics, Hospitals, Germany, Questionnaire survey, Empirical research

## Abstract

Nearly every eighth German hospital faces an elevated risk of bankruptcy. An inappropriate use of inventory management practices is among the causes. Hospitals suffer from demand and lead time uncertainty, and the current COVID-19 pandemic worsened the plight. The popular business logistics concept of risk pooling has been shown to reduce these uncertainties in industry and trade, but has been neglected as a variability reduction method in healthcare operations research and practice. Based on a survey with 223 German hospitals, this study explores how ten risk pooling methods can be adapted and applied in the healthcare context to reduce economic losses while maintaining a given service level. The results suggest that in general risk pooling may improve the economic situation of hospitals and, in particular, inventory pooling, transshipments, and product substitution for medications and consumer goods are the most effective methods in the healthcare context, while form postponement may be unsuitable for hospitals due to the required efforts, delay in treatments, and liability issues. The application of risk pooling in healthcare requires willingness to exchange information and to cooperate, adequate IT infrastructure, compatibility, adherence to healthcare laws and regulations, and securing the immediate treatment of emergencies. Compared to manufacturing and trading companies, hospitals seem to currently neglect the variability reducing effect of risk pooling.

## Introduction

The economic situation of German hospitals is difficult due to a decline in the number of inpatients, outpatient/inpatient-based rather than holistic remuneration systems, consolidation processes, unwillingness of hospital groups to compensate losses of individual members, and managerial mistakes (Augurzky et al. 2019; Telgheder 2019). More than every fourth German hospital group incurs losses, nearly every eighth hospital faces an elevated risk of bankruptcy, and the current COVID-19 pandemic is putting additional strain on hospitals (Augurzky et al. 2019; Lösch 2020).

Increasing costs, quality concerns, and challenges in inventory management lead to an increasing importance of logistics to improve efficiency and effectiveness in the healthcare context (Kritchanchai et al. 2018; Minvielle 2018). Jacobs and Chase (2020) report that the average inventory for a medium size hospital is $3.5 million, which represents 5–15% of current assets and 2–4% of total assets. Notwithstanding such problems, logistics culture and qualification still need to be anchored in hospitals (Benzidia et al. 2016; Ageron et al. 2018).

From the above it appears evident that applying business and logistics practices to hospitals may be beneficial. Hospitals struggle with demand uncertainty from the patient side (Nguyen et al. 2017; Ageron et al. 2018) and supply uncertainty (Zepeda et al., 2016, Dias et al. 2018). Gittell et al. (2000) argue that the level of uncertainty they experience may be much higher than in manufacturing organizations. This uncertainty can lead to excess capacity and higher costs (Almeida and Cima 2015).

Risk pooling has been shown to enable to reduce those uncertainties caused by variability and thus to lower costs for a given service level, increase the service level for given costs, or a combination of both in manufacturing and trading companies (Simchi-Levi et al. 2008; Cachon and Terwiesch 2019; Chopra and Meindl 2019). In general, healthcare operations management rarely considers risk pooling, and in the few cases different assumptions of optimization models are typically used (e.g. only one specific method, one specific item, benefits not related to variability-reduction). Only Joustra et al. (2010), Zepeda et al. (2014, 2016), and Stanger et al. (2013) explicitly examine risk pooling as a variability reducing method in healthcare.

This paper aims to complement previous research by adopting a wider perspective in the exploration of risk pooling methods’ adoption in the healthcare context: It investigates the perceptions of medical and operations management staff towards the application and applicability of ten risk pooling methods to medications, consumer goods, capital goods, examinations and treatments as well as their interrelationships in German hospitals. This research intends to contribute to the literature by clarifying the scope of risk pooling application possibilities and restrictions in hospitals and its cost reducing potential for a given service level.

Thus, this paper addresses the following research question:

*How can risk pooling be adapted and applied in the healthcare context to reduce economic losses while maintaining a given service level?*

To this aim the paper starts from the ten risk pooling methods identified by Oeser (2015) and analyzes the perceptions of medical and operations management staff regarding their adoption to medications, consumer goods, capital goods, examinations and treatments in a sample of 223 German hospitals.

The remainder of this paper is structured as follows: Sect. 2 reviews the fragmented literature on risk pooling methods in healthcare. Section 3 justifies and explains our survey approach. The survey data analysis and findings follow in Sect. 4, while theoretical and managerial implications are derived in Sect. 5. Section 6 concludes this paper highlighting its main contributions and avenues for further research.

## Theoretical background on risk pooling methods in healthcare

In business logistics, risk pooling refers to consolidating individual demand and/or lead time variabilities by aggregating demands and/or lead times in order to reduce the total variability they form and thus uncertainty and risk (Oeser 2015).

In demand pooling stochastic demands are aggregated so that above and below average demands can balance each other (Chen and Chen 2003). Demands may be aggregated across materials, products, locations, time (Simchi-Levi et al. 2008), and customers (Mc Guire 2015).

Lead-time pooling aggregates stochastic lead times, so that premature deliveries can compensate late ones and safety stocks and stockouts can be reduced (Evers 1999).

Oeser (2015) reviews the literature on risk pooling in business logistics and identifies ten risk pooling methods (Table 1).

**Table 1:** Ten risk pooling methods described (cf. Oeser 2015)

Value chain activity most addressed	Risk pooling method	Description
Storage	Inventory pooling	It combines stocks through physical centralization or selective stock-keeping to reduce inventory holding and stockout costs. By combining inventories, stochastic demands are aggregated, as they are satisfied by the consolidated inventory, and their fluctuations can balance each other
Transportation	Virtual pooling	It allows an organization’s location to view the inventory of other own or third-party locations using information and communication technologies and to access this inventory − e.g., in the event of a shortage − by drop-shipping or cross-filling, which corresponds to transshipments. Demands are aggregated across these locations so that their fluctuations can balance each other
	Transshipments	They refer to inventory transfers between locations (e.g., in case of a stockout). They lead to demand pooling across all locations, since alternative locations can satisfy demands, and lead time pooling, since the entire system has the option of partially replenishing inventory
Procurement	Order pooling or centralized ordering	It places joint orders for different locations and subsequently allocates them to the requisitioners based on current demand information. The inventory allocation decision is postponed and stochastic demands can be treated in an aggregate form until it is made. Demand fluctuations and forecast errors can balance each other, so that inventories and stockouts can be reduced
	Order splitting	It distributes an order to several suppliers or to several deliveries, treatments, or examinations. One order and thus its lead time is divided into several orders or deliveries and their respective lead times, so that the fluctuations of these lead times can balance each other
Production	Component commonality	It uses identical instead of individual parts for several medications, consumer goods, capital goods, treatments, or examinations. The demand for the individual parts is aggregated to the demand for fewer generic parts or one generic part, so that the variability of the demand for parts can be reduced
	Postponement	It delays decisions in the supply chain, maybe also in treatments and examinations. Due to the shortened forecast horizon and an aggregated forecast, more accurate information can be used. Aggregate forecasts are usually more accurate than disaggregate ones due to the statistical balancing effects of risk pooling. Postponement allows products to move downward the supply chain in a generic form longer and to differentiate them to customized products later according to more current demand information. At the preceding supply chain levels before the differentiation, the demand for the individual products is aggregated to the demand for the generic product, which fluctuates less strongly, since the stochastic fluctuations of the individual demands balance each other to a certain extent
	Capacity pooling	It combines production, service or storage capacities of several facilities. Without pooling, every facility only meets its demand with its own capacity. With pooling, the demands are aggregated and satisfied with the combined capacity. A higher level of service with the same capacity or the same level of service with less capacity can be offered. Capacity flexibility allows capacity shifts to high-turnover products in order to avoid lost sales
Distribution	Product pooling	It combines different product designs into one universal design (standardization) or reduces the number of product variants or stock keeping units, so that requests that were previously satisfied with their own product design are now served with fewer ones. The demands for the various products are aggregated to the demand for the universal design or the reduced number of stock keeping units, which fluctuates less thanks to risk pooling
	Product substitution	Customers are prompted to buy an alternative product because the originally requested product is out of stock or although it is in stock (in the case of demand reshape). Substitution allows to aggregate the demand for substitutable parts, products or services

While risk pooling has been extensively analyzed for industrial and trading companies (e.g. Johnston 2014; Wiengarten et al. 2017; Cachon and Terwiesch 2019), healthcare research on it still seems fragmented (see Table 2).

**Table 2:** Scientific research on risk pooling methods in healthcare

Risk pooling method	Source	Research country	Research method	Results
Inventory pooling	Hosseinifard and Abbasi (2018)	Australia	Two echelon blood inventory model, numerical study	Centralizing blood inventories from seven to three hospitals decreased outdate by 21% and shortage by 40%
Virtual pooling Transshipments Order pooling	Zepeda et al. (2014)	U.S.	Empirical examination	Weaker supply chain infrastructure and greater service mix uncertainty increase inventories System affiliation (common ownership of several hospitals and sharing supplies) can reduce supply chain risk and achieve scale advantages for small hospitals Purchasing groups by themselves do not necessarily reduce supply chain risk
Virtual pooling Transshipments	Zepeda et al. (2016)	U.S.	Econometric model	Local lateral emergency transshipments of supplies between hospitals outperform vertical integration with suppliers (obtaining inventory from higher levels) especially if the logistics infrastructure is weak
Transshipments	Stanger et al. (2013)	U.K.	Institutional theory, case studies and follow-up survey with 16 hospital transfusion laboratories	Lateral transshipments (on the same echelon) of perishable blood between UK hospitals can increase efficiency and effectiveness
Transshipments	Fattore et al. (2014)	Italy	Descriptive analysis of 2009 data on 11,531 National Health Service admissions	Interregional mobility requires financial and relational resources. Private hospitals are more likely to admit non-resident patients
Transshipments	Wang and Ma (2015)	China	Age-based transshipment model with two preference selection methods for transshipping blood units during a blood shortage, simulation	A first-in-first-transship policy can reduce the expired rate more efficiently than a quantity-based model. However, the transshipment decision may increase the expired and overstock ratio after the blood shortage
Transshipments	Ageron et al. (2018)		Editorial to the special issue on Healthcare Logistics and Supply Chain	Inter-hospital transshipments of patients demand effective logistics of the participating hospitals, transfusion centers, and laboratories and may carry the risk of complications and information loss
Transshipments Postponement	Parvin et al. (2018)	Malawi	Integration of strategic-level and tactical-level models (two-stage stochastic programming and Markov decision process), numerical study of 290 facilities	Minimization of transportation and shortage costs for malaria medication in a three-tier centralized distribution system in Malawi using lateral transshipments between clinics and delayed shipments by holding back inventory at a higher echelon
Order pooling	Kim and Skordis-Worrall (2017)	U.K.	Generalized linear model	Voluntary pooled procurement can reduce drug prices
Order pooling	Baldi and Vannoni (2017)	Italy	Econometric models, data on tender prices of selected drugs for hospital usage provided by a sample of 52 Italian local health service providers between 2009 and 2012	Centralized public procurement leads to lower drug prices, especially in areas with lower institutional quality or higher corruption
Order pooling Transshipments	Adida et al. (2011)	U.S.	Game theory (non-cooperative strategic game)	Nash equilibrium is proven for joint inventory stockpiling of medical supplies for groups of hospitals before a disaster Increasing the shortage penalty or decreasing the procurement cost increases the equilibrium stockpile closer to the system optimal stockpile
Postponement	Vasquez et al. (2017)	U.S.	Clinical study: intramammary antimicrobial treatment of all cases of clinical mastitis with a selective treatment protocol based on 24-h culture results	Delayed pathogen-based treatment could reduce the number of hospital days compared to immediate blanked treatment of clinical mastitis in a dairy herd
Capacity pooling	Reymondon (2008)	France	Computer aided system design, optimization model	Methodology for grouping and sharing resources in sterilization of reusable medical devices that minimizes process and storage costs
Capacity pooling	Joustra et al. (2010)	The Netherlands	Queuing theory, discrete event simulation, realistic case	For one kind of service capacity pooling reduces waiting time and/or total capacity required. Pooling urgent and regular patient queues, however, may increase waiting times for urgent patients, if the utilization by them is small and by regular patients is large. Separating these queues may decrease the required capacity for given waiting time targets for both patient groups
Product substitution	Alkhuzaee et al. (2016)	Saudi Arabia	Face-to-face questionnaire survey (*n* = 121)	Pharmacists conduct generic medicines substitution due to costs and patient requests
Product substitution Inventory pooling	Saedi et al. (2016)	U.S.	Stochastic inventory optimization model to minimize the effect of drug shortages under uncertain disruptions and demand	Drugs may be substituted in case of supply disruptions. The substitute may not be as effective The model performs better than current policies except for holding cost because of the current low space utilization Hospitals likely benefit from inventory pooling
Product substitution	Diamant et al. (2018)	Canada	Base-stock inventory models, one considering stockout-based substitution	The number of reusable instrument sets held in inventory may be reduced if on-site sterilization techniques (e.g., flash sterilization) are used Open-surgery is typically performed when no laparoscopic instruments are available. In addition, flash sterilization may be used instead of off-site sterilization or the operation may be rescheduled
Product substitution	Ma et al. (2019)	China	Mixed integer programming model	Considering ABO/*Rh*(D)-compatible blood substitution can increase the efficiency of emergency blood allocation while lowering blood shortage

Zepeda et al. (2014, 2016) and Stanger et al. (2013) explicitly focus on risk pooling or pooling in short. Diamant et al. (2018) and Ma et al. (2019) describe the risk pooling effect without mentioning the term. Joustra et al. (2010) analyze the effect of pooling queues of urgent and regular patients on waiting times and capacities required.

Reymondon et al. (2008), Fattore et al. (2014), Alkhuzaee et al. (2016), Baldi and Vannoni (2017), Kim and Skordis-Worrall (2017) and Vasquez et al. (2017) do not consider risk pooling and the variability reducing balancing effect of the respective method. For the remaining sources risk pooling is not the focus of their optimization models.

Adida et al. (2011) explicitly mention risk pooling, but their model does not consider it, as they focus on the total shortage and associated cost as well as the stochasticity of total demand for a group of hospitals and not each individual hospital forming this group. In their model, the total penalty cost is allocated to the individual hospitals proportionally.

In manufacturing, postponement has been used as a risk-pooling method for a long time to enable producing a generic product cost-efficiently in mass production and customizing it later according to individual customer wishes (mass customization) (Wiengarten et al. 2017). For healthcare, Mannion and Exworthy (2018, p. 572) discuss the “under-researched area” of standardization and (mass) customization from an institutional logics perspective, but not from a risk-pooling perspective.

Most research on risk pooling in healthcare seems to have been conducted outside Europe (especially in the U.S.) and we found no study in Germany. Most studies consider transshipments, order pooling, and product substitution, while order splitting, component commonality, and product pooling seem to be neglected. Mostly different terms are used, which could be subsumed under the respective risk pooling method named in Table 2. Most studies focus on a single risk pooling method, a single item (blood, medication or medical supply) or a model, especially an optimization model.

## Method

To address the research question this study uses data on the medical and operations management staff perception of risk pooling application and applicability in a sample of German hospitals. By quantitatively describing the characteristics and tendencies of the sample this research seeks to identify general patterns that can be extended to a larger population of hospitals.

In line with Robson (2002) and Babbie (2007) explorative and descriptive research is appropriate here to gain a better understanding of risk pooling application and applicability, interrelationships and restrictions, since risk pooling is a relatively new research area in healthcare operations management, especially its variability reduction perspective, as evidenced by the scarce and fragmented literature.

German hospitals offer a high quality in terms of access and resources (OECD 2019). Therefore, they have been able to cope with the COVID-19 pandemic better than other countries so far (Augustin 2020), but struggle economically (Augurzky et al. 2019). Thus, they seem suitable to explore how risk pooling can be applied and adapted in hospitals to reduce demand and lead time variability.

We researched the e-mail addresses of the general and medical directors of every German hospital, contacted them via e-mail, and asked them to have the − in their opinion − key informants in their hospital answer our online questionnaire (see appendix). This ensured the competence of the respondents. Free-text answers and triangulation with quantitative external data made it possible for us to ascertain further that the respondents were knowledgeable about the investigated issues. For instance, external data support that private hospitals indeed perform better economically than public ones (Augurzky et al. 2019) and that hospitals severely suffer from supply uncertainty for medications (Müller 2019).

Afterwards, 223 participants completed the questionnaire from January 30, 2017 until March 30, 2017. On February 28, 2017, 97 surveyees had participated and a follow-up e-mail was sent asking non-responders to fill out the questionnaire. Due to the inertia of health organizations (Wang et al. 2015), this survey data should still be up to date.

One hundred thirty-three participants reported to be non-medical staff, 62 to be medical staff. Only 107 participants gave their specific job titles: 18 chief physicians, 12 managing directors, 10 medical directors, 9 nurses, 8 commercial directors, 7 commercial department employees, 7 administrative directors, 7 logistics directors, 4 assistants, 3 senior physicians, 2 chief physician secretaries, 2 quality management officers, 2 managers, 1 head of medical controlling, 1 boss, 1 owner, 1 purchasing manager, 1 coordinator, 1 head of human resources for the complete network, 1 head of outgoing goods, 1 manager medicine and materials, 1 nursing director, 1 authorized officer, 1 process manager, 1 assistant to the medical director, 1 clerk for purchasing and logistics, 1 employee from the general unit administration and legal matters, 1 ward doctor, and 1 chairman of the clinic board.

Fourty-one non-responders replied to us by e-mail that they do not find the time to answer the survey or that their hospital policy is not to participate in surveys on principle, as they receive many such requests. Therefore, it is assumed that the reason for the nonresponse is not associated with the measured statistical values and therefore it does not affect the quality of the survey’s results (Groves et al. 2004).

The answers before and after the follow-up e-mail do not differ statistically significantly (*p* > 0.05) in t-tests for the quantitative variables (number of employees, number of beds and economic situation) and chi-square tests or, where appropriate, Fisher’s exact test for the nominal variables. Thus, a late-response bias (Mentzer and Flint 1997) and, assuming that the answers of late and non-respondents are similar (Armstrong and Overton 1977), a non-response bias are unlikely.

Participants were first asked to give general information (public, non-profit, or private hospital; number of employees; number of beds; the participant’s position; the participant’s assessment of the hospital’s economic situation). Afterwards they were asked if they consider demands for as well as lead times of medications, consumer goods, capital goods, examinations, or treatments to be uncertain or fluctuating.

Then participants were given definitions and examples of each risk pooling method and asked in yes-no questions if the methods are currently applied or could be applied in their hospitals to each good and service category and what restrictions they perceived in applying these methods in free-text fields. Thus, the present study collected and analyzed the estimations, perceptions, and opinions of the respondents on the application and applicability of risk pooling methods in their hospital. Therefore, it regards the perceived application and perceived applicability of risk pooling, although − to facilitate readability − we simply use the terms application and applicability in the following.

## Data analysis and findings

In order to answer the research question the following research items (RIs) are analyzed in this section:

1. What share of German hospitals applies the ten risk pooling methods to medications, consumer goods, capital goods, examinations, and treatments (RI1)?
2. What share considers the ten risk pooling methods applicable to the above items (RI2)?
3. Does the application and applicability differ between public, non-profit and private hospitals (RI3)?
4. Does the application differ between large and small hospitals (RI4)?
5. Does the applicability appraisal differ by the respondents’ job position (RI5)?
6. Is the application of different risk pooling methods correlated (RI6)?
7. Is demand and lead time uncertainty correlated with the application of risk pooling (RI7)?
8. Is the hospitals’ economic situation correlated with the application of risk pooling (RI8)?
9. What restrictions exist for the application of risk pooling in hospitals (RI9)?

### Application of risk pooling methods (RI1)

**Fig. 1:**
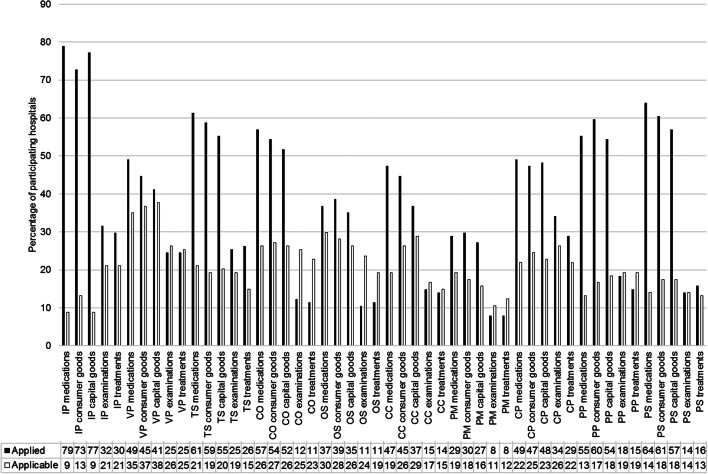
Application and applicability of risk pooling in German hospitals. Notes: Inventory pooling (IP), virtual pooling (VP), transshipments (TS), centralized ordering (CO), order splitting (OS), component commonality (CC), postponement (PM), capacity pooling (CP), product pooling (PP), product substitution (PS)

In decreasing order of the mean percentage of hospitals applying each risk pooling method across all goods and services, German hospitals seem to use inventory pooling (58.1%), transshipments (45.4%), and product substitution (42.3%), followed by capacity pooling (41.6%), product pooling (40.5%), centralized ordering (37.4%) and virtual pooling (36.8%). Component commonality (31.6%), order splitting (26.5%) and postponement (20.3%) are less applied (Fig. 1).

Risk pooling seems to be applied the most to medications (except order splitting, postponement, and product pooling to consumer goods), the second most to consumer goods (except inventory pooling and capacity pooling to capital goods). It appears to be less applied to examinations and treatments. In particular, it is scarcely applied to treatments, except for transshipments, order splitting and product substitution, which an even lower percentage of hospitals uses with regard to examinations.

### Applicability of risk pooling methods (RI2)

In addition to the current risk pooling usage, virtual pooling of capital goods (37.7%), consumer goods (36.8%), and medications (35.1%), splitting of medication orders (29.8%), and component commonality regarding capital goods (28.9%) seem to be most applicable in healthcare prospectively. Order splitting (28.1%) and centralized ordering (27.2%) of consumer goods follow in the order of decreasing applicability. Virtual pooling regarding examinations, centralized ordering of medications and capital goods, splitting capital goods orders, component commonality regarding consumer goods, and pooling examination capacities are each considered applicable by 26.3% of the sample (Fig. 1).

### Application and applicability by hospital type (RI3)

Based on chi-squared tests, non-profit hospitals appear to apply virtual pooling to consumer goods (Goodman and Kruskal’s tau (*τ*) = 0.107, *p* = 0.024) and capacity pooling for examinations (*τ* = 0.153, *p* = 0.002) more. Public hospitals seem to apply inventory pooling to capital goods (*τ* = 0.101, *p* = 0.007), centralized ordering for examinations (*τ* = 0.092, *p* = 0.022), inventory pooling to consumer goods (*τ* = 0.078, *p* = 0.018) and virtual pooling to capital goods (*τ* = 0.095, *p* = 0.042) less. The second to fourth relationship stay significant after post-hoc testing with a Bonferroni correction based on adjusted standardized residuals (cf. Beasley and Schumacker 1995; García-Pérez and Núñez-Antón 2003), the second one is the strongest.

More participants from non-profit hospitals consider capacity pooling for examinations (*τ* = 0.146, *p* = 0.005) and capital goods (*τ* = 0.140, *p* = 0.021) and order splitting for examinations (*τ* = 0.069, *p* = 0.032) applicable. The first relationship stays significant after post-hoc testing with Bonferroni correction. Capacity pooling for treatments is considered less applicable by participants from private hospitals (*τ* = 0.104, *p* = 0.018). Judging by Goodman and Kruskal’s tau these correlations are rather weak, which often happens for bivariate distributions of social scientific data, because social and economic interrelations may not be transparent at first glance, but multidimensional, flexible, and versatile (Müller-Benedict 2007).

### Application and applicability by hospital size (RI4)

The ratio variables of number of employees and number of beds are considered as indicators for hospital size. Point biserial correlation coefficients (*r*_*pb*_) are calculated considering these ratio variables as independent and the dichotomous risk pooling application and applicability variables as dependent (cf. Warner 2008). In this case *r*_*pb*_ corresponds to the Pearson product-moment correlation coefficient *r* (Warner 2008).

A hospital is more likely to experience uncertain examination lead times, the larger it is in terms of number of employees (*r*_*pb*_ = 0.273, *p* = 0.006) and number of beds (*r*_*pb*_ = 0.226, *p* = 0.018). Consequently, larger hospitals seem to focus on risk pooling with respect to examinations and to the subsequent treatments: Hospital size is statistically significantly and positively correlated with the application of product substitution in examinations (*r*_*pb*_ = 0.237, *p* = 0.028 for the number of employees) and treatments (*r*_*pb*_ = 0.278, *p* = 0.000 for the number of employees; *r*_*pb*_ = 0.220, *p* = 0.033 for the number of beds).

The larger the hospital is, the more its survey participant considers the following risk pooling methods applicable: transshipments with regard to examinations (*r*_*pb*_ = 0.233, *p* = 0.041 for the number of employees) and treatments (*r*_*pb*_ = 0.294, *p* = 0.011; *r*_*pb*_ = 0.223, *p* = 0.047), component commonality in examinations (*r*_*pb*_ = 0.286, *p* = 0.007; *r*_*pb*_ = 0.224, *p* = 0.030) and treatments (*r*_*pb*_ = 0.321, *p* = 0.002; *r*_*pb*_ = 0.252, *p* = 0.014), and postponement in examinations (*r*_*pb*_ = 0.328, *p* = 0.001; *r*_*pb*_ = 0.235, p = 0.018) and treatments (*r*_*pb*_ = 0.338, *p* = 0.001; *r*_*pb*_ = 0.292, *p* = 0.003).

### Applicability by job position (RI5)

The job position may influence the applicability assessment regarding risk pooling based on chi-squared tests. Survey participants working in administrative positions consider centralized ordering of medications (*τ* = 0.199, p = 0.011), capital goods (*p* = 0.022) and consumer goods (*p* = 0.039), transshipments of consumer goods (*p* = 0.026) and product substitution of capital goods (*p* = 0.035) more applicable. The first relationship is the strongest and stays statistically significant after post-hoc testing with Bonferroni correction, Fisher’s exact test value (2, 45) = 8.792, *p* = 0.011.

### Correlations between risk pooling methods (RI6)

The application of a particular risk pooling method to medications, consumer goods, and capital goods is often mutually strongly and highly significantly correlated. For example, the order splitting of medications shows the second highest correlation to order splitting of consumer goods (*r* = 0.974, *p* = 0.000). Substitution of capital and consumer goods shows the third highest correlation (*r* = 0.973, *p* = 0.000). The application of the risk pooling methods to examinations and treatments is also often strongly and highly significantly correlated. For example, the postponement of examinations is perfectly positively correlated with the postponement of treatments (*r* = 1.000, *p* = 0.000): Examinations cannot be postponed, unless the following treatment is postponed as well. Using common components in examinations may entail the use of common components in treatments (*r* = 0.964, *p* = 0.000, fourth highest correlation).

This shows that the application of risk pooling to physical goods and services seems to be perceived as mostly detached. However, the application to different goods may go hand in hand, as does the application to different services. Still pooling of capital goods capacities may entail capacity pooling for examinations (*r* = 0.579, *p* = 0.000) and treatments (*r* = 0.576, *p* = 0.000).

In decreasing order of strength of correlation, centralized ordering and component commonality, inventory pooling and virtual pooling, centralized ordering and capacity pooling, order splitting and component commonality, virtual pooling and centralized ordering, virtual pooling and transshipments are associated. For instance, the following risk pooling applications are highly statistically significantly correlated (*p* < 0.0001): centralized ordering and component commonality in treatments (*r* = 0.804), inventory and virtual pooling of medications (*r* = 0.734), centralized ordering and capacity pooling with regard to consumer goods (*r* = 0.724), order splitting in examinations and component commonality in treatments and examinations (*r* = 0.687), order splitting and component commonality in treatments (*r* = 0.656), virtual pooling and centralized ordering of both capital goods (*r* = 0.679) and consumer goods (*r* = 0.678), and virtual pooling and transshipments of capital goods (*r* = 0.666).

Centralized ordering may go hand in hand with standardizing and using common capacities. Centralized ordering requires access to current inventory and demand data, which may be implemented via virtual pooling. Inventories may not have to be pooled physically, but could also be accessed via information and communication technologies. If the inventories are then needed physically at another location they may be transferred via transshipments.

Risk pooling in procurement (including common components) seems to be most strongly correlated with the application of risk pooling in production and storage. Demand pooling (virtual pooling and component commonality) and lead time pooling (transshipments and order splitting) may be used together to balance their disadvantages. The free-text answers confirmed this: One non-profit hospital with 188 beds does not use product substitution “because there is either central storage or one of the other hospitals is asked” (nurse). Thus, this hospital relies on other risk pooling methods (inventory pooling and transshipments). Others use centralized ordering and transshipments instead of product substitution (nurse in accident surgery, private hospital, 284 beds).

### Demand and lead time uncertainties and application of risk pooling methods (RI7)

The largest percentage of participants (39.1%; *n* = 202) considers the demands for treatments fluctuating or uncertain, followed by demands for medications (29.2%), examinations (27.7%), consumer goods (20.8%), and capital goods (17.8%) in order of decreasing percentages of participants.

Lead times for medications, examinations and treatments are assessed as fluctuating or uncertain by the largest share (25.7%; *n* = 191), followed by capital goods (25.1%) and consumer goods (22.5%).

A higher share of participants considers the demands for medications, examinations and treatments uncertain compared to their lead times. The opposite applies to consumer and capital goods.

The different types of hospitals differ statistically significantly in their assessments of the uncertainty of demand for medications, *χ2* (2, 184) = 7.464, *p* = 0.024, *τ* = 0.041. 48.7% of participants from private hospitals believe to suffer from demand uncertainty for medications, only 24.7% from public and 27.9% from non-profit ones (*p* = 0.00696 < *p* = 0.00833 instead of 0.05 for 6 comparisons in a Bonferroni correction). Thus, private hospitals may be more suitable candidates for demand pooling.

Only 16.7% of the administration (*p* = 0.0001), but 44.8% of doctors (*p* = 0.0005) and 44.4% of nurses (non-significant Bonferroni corrected *p* = 0.2659) see medication lead times as uncertain, *χ2* (2, 163) = 15.393, *p* = 0.001, *τ* = 0.094. Maybe doctors and nurses are closer to the point of usage to experience uncertain medication lead times.

Uncertainties are not strongly associated with the application of risk pooling methods. There are only six highly statistically significant (*p* < 0.01) correlations between uncertainties and the application of risk pooling methods.

The strongest and most statistically significant correlations exist between uncertain demand for treatments and virtual pooling of consumer goods (*r* = 0.324, *p* = 0.006) and postponement of capital goods (*r* = 0.289, *p* = 0.004).

This may suggest that the participants do not associate risk pooling methods strongly with mitigating uncertainties. Hospitals may apply the risk pooling methods for other benefits such as centralized ordering for economies of scale. However, they may also reap “statistical economies of scale” (Eppen 1979, p. 498, Özer 2003, p. 269) from its application.

Uncertain lead times for capital goods are correlated with virtual pooling of medications (*r* = 0.310, *p* = 0.007), product pooling of capital goods (*r* = 0.279, *p* = 0.007), and order splitting for capital goods (*r* = 0.275, *p* = 0.011). Uncertain lead times for consumer goods are associated with pooling treatment capacities (*r* = 0.281, *p* = 0.008) and product pooling of consumer goods (*r* = 266, *p* = 0.009). This shows that uncertain lead times are not necessarily associated with lead time pooling.

### Economic situation and application of risk pooling methods (RI8)

The participants rated the economic situation of their hospitals on a six-point likert scale, which corresponds to the German school grading system where 1 is the best and 6 the worst. On average, they considered the economic situation of their hospitals as satisfactory with a mean of 2.87 and an empirical standard deviation of 1.26.

There was a significant effect of the type of hospital on the assessment of the economic situation in a one-way ANOVA, *F*(2, 177) = 5.488, *p* = 0.005, with an appropriately non-significant Levene’s test (*p* = 0.927). Games-Howell post hoc tests showed that participants from public hospitals and non-profit hospitals assessed the economic situation of their hospitals (mean = 2.96 and 2.88) statistically significantly (*p* = 0.006 and 0.017) worse than participants from private hospitals (mean = 2.24). Nearly 6% of the variability in economic situation is accounted for by group membership (partial eta squared = 0.058). The Krankenhaus Rating Report 2019 supports that private hospitals indeed perform better economically than public ones (Augurzky et al. 2019).

Participants whose hospitals apply risk pooling seem to predominantly assess their hospital’s economic situation better: 33 of the 50 risk pooling variables show a negative correlation with the economic situation variable. This correlation is statistically significant only for inventory pooling re medications (Kendall rank correlation coefficient tau-b (*τ-b*) = −0.193, exact significance *p* = 0.037), consumer goods (*τ-b* = −0,185, *p* = 0.049), treatments (*τ-b* = −0.234, *p* = 0.020) and examinations (*τ-b* = −0.234, *p* = 0.022).

In accordance with De Vaus (2013), we treated the dichotomous risk pooling application variables as being ordinal in order to analyze their associations with the ordinal variable economic situation and retain the ordinal information of this variable. The Kendall rank correlation coefficient tau-b was chosen for its conservativeness (Blaikie 2003) and robustness to outliers (Croux and Dehon 2010).

Product substitution in treatments (*τ* = 0.308, *p* = 0.001) and examinations (*τ* = 0.228, *p* = 0.019) show the only statistically significant positive correlations with the economic situation variable. Chi-square post-hoc testing with Bonferroni correction shows that hospitals rating their economic situation with a five tend to apply product substitution re treatments (*p* = 0.0000 < 0.0042 instead of 0.05 for our 12 comparisons) and examinations (*p* = 0.0001) more than expected.

### Restrictions (RI9)

In 150 free-text responses (on average 15 per risk pooling method), 51 participants gave further information on the application and applicability of risk pooling in German hospitals. Costs of implementing and using risk pooling have to be weighed against the benefits of reduced variability and thus potentially lower inventory and capacity costs and lower stockouts. Advantages and disadvantages of the different risk pooling methods in business logistics have already been analyzed by Oeser (2015). Therefore, here we consider the peculiarities in hospital logistics. Compared to business logistics, a stockout in hospital logistics does not lead to a lost sale but maybe prolonged suffering or even death (Jacobs and Chase 2020). Restrictions to implementing the risk pooling methods in German hospitals are highlighted in Table 3.

**Table 3:** Risk pooling restrictions in hospitals

Risk pooling method	Restriction	Example quotation (source)
Inventory pooling	Possibly longer transportation, therefore decentral emergency/safety stock with rapid replenishment for critical items and fast movers needed	“After a centralization, logistics had deteriorated due to longer routes and a regionalization of the supply structures” (senior physician, private hospital, 400 employees)
		“Standard and bulky items should be stored locally” (logistics manager, public hospital, 2200 employees)
		“Standard items should be stored in every examination and treatment room. A central depot could only function as a backup and replenishment warehouse for them” (logistics manager, private hospital, 1800 employees)
	Optimal pooling location	“Choosing the right location for a central warehouse is crucial for a positive effect” (managing director, non-profit hospital, 136 employees)
Virtual pooling	Handy IT infrastructure required	“We sometimes do this by phone, as the IT systems are unwieldy and take a lot of time (senior physician, public hospital, 13,200 employees)
		“We use an ordering system via a central pharmacy and a warehouse, which does not allow to view actual stocks or temporal valences (process manager, public hospital, 80 employees)
	Willingness to exchange information	Senior physician, public hospital, 13,200 employees
	Pharmacy Act for medications, quality control	“This is only feasible for medications and the Pharmacy Act has to be followed” (department of economics and supply, public hospital, 860 employees)
		“This may lead to expired inventories and mistakes in active ingredients” (manager in charge of medicine and materials)
	Electronic booking and telemedicine for examinations and treatments	Electronic booking of services from other areas, treatment requirements and scheduling is possible and sensible (logistics manager, non-profit hospital, 1800 employees; process manager, public hospital, 80 employees). “For treatments and examinations only useful to a limited extent (telemedicine)” (medical director, non-profit hospital, 1300 employees)
Transshipments	High transportation cost, especially for transshipping patients	Chief physician and medical director, non-profit hospital, 1000 employees
	Appropriate transportation mode	“The patient’s state of health and the appropriate means of transport have to be considered” (managing director, non-profit hospital, 136 employees)
	Only in case of shortages	“This is only possible for non-time-critical standard articles. Supplies for acute needs have to be held in the area and sometimes in individual rooms” (logistics manager, private hospital, 1800 employees)
		“Usually examination and treatment services are provided in your own hospital; if this cannot be done, the patient has to be transferred to the nearest health facility that can provide these services. Exceptions are consoles or services from the tertiary sector of a hospital such as laboratory, radiology or other imaging procedures, physiotherapy, patient transport, to name just a few examples” (managing director, public hospital, 1,320 employees)
Centralized ordering	Willingness to cooperate, coordination effort, heterogeneous needs and wants	“Our logistics center supplies 150 hospitals, which form a purchasing group. The individual hospitals benefit from cheaper purchasing conditions, but have to agree on the purchased products” (manager)
		“Each department still orders for itself because of different needs and wants” (senior physician, public university hospital, 13,200 employees)
Order splitting	Current commitment to suppliers, incompatibility of products	We have many different suppliers (multi-sourcing) and change products regularly because of the price (senior physician, public hospital, 13,200 employees; medical director, non-profit hospital, 350 beds)
		“We have only one provider for a specific product (single-sourcing), as we are tied by agreements and some products are incompatible” (clerk for purchasing and logistics, non-profit hospital, 497 beds)
	Not applicable to examinations and treatments	“I see order splitting for examinations and treatments critical due to more efforts” (department of economics and supply, public hospital, 860 employees) and “specialization in certain illnesses” (secretary to the chief physician, public hospital, 55 beds)
Component commonality	Standardization effort especially for examinations and treatments	“We came up with a list of standard medicine (400 to 600 different medications). This standardization is welcomed as different pharmaceutical companies use different packaging, shapes, and colors for the same medicine, which confuses elder patients in particular” (medical director, non-profit hospital, 350 beds)
		“Standardized devices from a manufacturer could be procured in order to reduce purchase prices, training and maintenance costs. Standardized processes and protocols would be too expensive for every illness (senior physician, public hospital, 13,200 employees)
	Incompatibility	“This could be hindered by lacking compatibility between medical products” (clerk for purchasing and logistics, non-profit hospital, 497 beds)
Postponement	Immediate treatment of emergencies	“Postponing service, examination and treatment: Not possible in a hospital! There is a care mandate and sick people need to be cared for and treated as quickly as possible!” (administration, non-profit hospital, 875 employees)
		“Emergencies need to be dealt with immediately, other cases can be postponed to obtain more information from experts” (senior physician, public hospital, 13,200 employees)
	Limited applicability of form postponement to Z goods with highly fluctuating demand	“Makes little sense in the supply with medical goods” (medical director, non-profit hospital, 310 beds; senior physician, public hospital, 13,200 employees; medical director, public hospital, 1300 employees)
		“We produce rare concentrations and combinations ourselves locally” (medical director, non-profit hospital, 350 beds)
		“Our logistics center prepackages pills for the patients in a pharmacy. Therefore, sachets of pills are provided for the patients, delivered to the hospitals every day and given to the patients. The nurses no longer have to sort the pallets” (manager)
	Place postponement	“Materials are ordered and stored centrally, until a need arises in different departments; this helps to act faster in emergencies” (nurse, 1200 employees)
	Hygiene guidelines, Medical Products Act, medicines regulations, liability	Purchasing generic medical goods and configuring them according to current requirements makes the hospital a manufacturer, which has to observe hygiene guidelines, the Medical Products Act, and the medicines regulations and becomes liable (medical director, public hospital, 571 employees; chief physician and medical director, non-profit hospital, 1000 employees; head of the department of economics and supply, public hospital, 860 employees; medical director, public hospital, 1700 employees; doctor and administration, public hospital, 10,400 employees)
	Risk of confusion	“Mixing and cutting to format of medical products poses the risk of confusion, especially under time pressure” (senior physician, public hospital, 13,200 employees)
		“If tablets are split, the unpacked medications may not be assignable anymore” (head of the department of economics and supply, public hospital, 860 employees)
	High effort of form postponement	Too time-consuming, resource-consuming and personnel-intensive (process manager, public hospital, 80 employees; medical director, public hospital, 1700 employees; senior physician, public hospital, 230 employees), e.g. “when cutting and folding compresses to the right format” (process manager, public hospital, 80 employees), “when packaging split tablets or diluting cleaning agents possibly with additional personal protective equipment” (head of the department of economics and supply, public hospital, 860 employees). “Buying the corresponding goods is usually cheaper” (head of the department of economics and supply, public hospital, 860 employees; assistant to the operations manager, public hospital, 1180 employees)
Capacity pooling	Willingness to collaborate	“The competitive thinking between hospitals makes collaborations impossible”
	Longer travel times for patients	“Hospitals should focus on specific treatments, e.g. one hospital on bone surgery and one on organ surgery” (senior physician, public hospital, 900 employees)
		“Centralization of treatments leads to longer travel times for patients. Patients should be treated close to their home in their interest and the interest of their relatives” (medical director, public hospital, 1300 employees)
	Specific procedures need to be adapted	“The centralization of treatments and examinations depends on defined process sequences and the use of digital systems (e.g. order / entry systems)” (medical director, public hospital, 1300 employees)
		“Each functional area has its own inventory and specific adapted procedures so that a common use is unfavorable” (private hospital, 1205 beds)
Product pooling	Current commitment to suppliers	“It is difficult to standardize, because we have agreements with specific suppliers” (clerk for purchasing and logistics, non-profit hospital, 497 beds)
	Preferences of doctors and patients	“Patients and doctors insist on specific medications and products” (head of the department of economics and supply, public hospital, 860 employees)
Product substitution	Incompatibility	“We use alternative products rarely due to a low compatibility” (clerk for purchasing and logistics, non-profit hospital, 497 beds)
	Possibly lower quality	“Substitutes may have a lower quality” (senior physician, public hospital, 230 employees)
	Flexibility required	“A high degree of flexibility is required in the medical field to react to delivery bottlenecks and stockouts quickly. As a rule, different manufacturers offer similar products, which can be used interchangeably without any problems due to the high degree of standardization. Cable or hose connections are also standardized, so that systems from different manufacturers are compatible with each other. Active ingredients are always the same, no matter which manufacturer produces the drug” (private hospital, 1205 beds)

Risk pooling requires willingness to exchange information and to cooperate between departments or hospitals and an IT infrastructure that facilitates this. Healthcare regulations, such as hygiene guidelines, the Medical Products Act, and the Pharmacy Act, and quality control have to be observed, which may make postponement unattractive. Incompatibility may hinder order splitting, component commonality, and product substitution. It must be ensured that emergencies can be treated immediately.

## Discussion

### Theoretical implications

#### Holistic research approach

Most previous research considers a single risk pooling method, a single item (blood, medication or medical supply) and optimization model. Only four scientific studies on risk pooling methods in healthcare focus on their variability reducing effect (see Table 2).

This paper complements this fragmented body of research, as it adopts a more holistic view exploring the application and applicability of ten risk pooling methods in German hospitals to medications, capital goods, consumer goods, examinations, and treatments as well as their interrelationships and restrictions. Considering these elements and their interactions together leads to a more integrated and realistic picture of the application and applicability of risk pooling in hospitals. It enables additional insights compared to the analysis of individual elements (Graman and Magazine 2006), such as which risk pooling applications seem to fit together and complement one another. Therefore, by shedding light on the “chemistry” of the holistic approach, the findings of the present study contribute to the research on the adoption of risk pooling and other operations management approaches (Graman and Magazine 2006; Boone et al. 2007; Oeser 2015) in healthcare.

#### Extension to examinations and treatments

While risk pooling has been analyzed extensively for goods in manufacturing and trading (e.g. Simchi-Levi et al. 2008; Oeser 2015; Cachon and Terwiesch 2019), the healthcare literature focusses on medications, blood, and medical supplies. This research checks the suitability of ten risk pooling methods for services to patients such as treatments and examinations. This contributes to our understanding of implementing efficiency methods in healthcare, which some healthcare professionals consider unsuitable for such services (Langabeer et al. 2009).

While risk pooling methods are applied to goods more than to healthcare services, capacity pooling (34.2% and 28.9%), inventory pooling (31.6% and 29.8%) and transshipments (25.4% and 26.3%) are used the most with regard to examinations and treatments respectively. Postponement of examinations and treatments is used the least by 7.9% of hospitals. The finding that risk pooling methods can be and are applied to examinations and treatments is a novel and original contribution of the present research.

Standardization of treatment processes can increase service quality and reduce medical mistakes (Boyer and Pronovost 2010; Andritsos and Tang 2014) and throughput time (Jha et al. 2016), but is applied and considered applicable by only 14% and 14.9% of our sample, as they are discouraged by the standardization effort. However, for larger hospitals this may still be a viable option, as they experience more uncertain examination lead times and therefore value component commonality in examinations and treatments more (cf. Sect. 4.4).

#### Restrictions of prolonged suffering and death

In business logistics, risk pooling methods may increase costs and service times and decrease product functionality, customer service, sales and profit in certain areas. Hospital logistics has to ensure that the application of risk pooling does not negatively affect the health of patients. While business logistics mainly considers the cost of implementing risk pooling methods, healthcare laws and regulations, information exchange, cooperation, and incompatibility seem to be the biggest obstacles in implementing risk pooling in hospitals. Although Mc Guire (2015) suggests many ways to apply form postponement to medical products, most participants consider it not suitable in hospitals, as they fear the postponement time and cost, delays in treatments, possibility of confusion, and liability issues.

#### Positive association with the economic situation

This research is the first to empirically show that applying risk pooling is mostly positively associated with the economic performance of hospitals. Previous research mainly modeled the effect of risk pooling on inventory and service levels, without building a bridge to the overall economic situation of an organization (Sect. 2; Oeser 2015), maybe because it is considered “operational hedging” (Van Mieghem 2007) and therefore the focus rests on operations. This paper also shows that risk pooling methods may differ in their suitability and association with the economic performance. For instance, product substitution in treatments and examinations could be economically disadvantageous for hospitals overall.

#### Complementarity of risk pooling methods

It is supported that demand pooling (virtual pooling and component commonality) and lead time pooling (transshipments and order splitting) may be used together to balance their disadvantages. While virtual pooling and component commonality can only reduce demand variability, order splitting can only lower lead-time variability and transshipments may decrease both types of variability.

#### Neglect of statistical economies of scale

The low correlation of uncertainties with the application of risk pooling methods suggests that hospitals may currently neglect the variability-reduction benefit of risk pooling like scientific studies on risk pooling methods in healthcare do (Table 2). This research can contribute to making decision makers aware of the additional risk-pooling benefit and of how to reap it.

For instance, many hospitals have formed purchasing groups in order to reduce purchase prices, but without taking advantage of the additional risk-pooling effect of order pooling. Hospitals may place joint orders instead of individual orders. With individual orders each hospital needs do deal with its own demand fluctuations and the place of delivery needs to be specified when placing the order. If the purchasing group places joint orders for (some of) its members, their stochastic demands are aggregated and can balance each other to a certain extend. The delivery destination decision is postponed and can be made according to more recent demand information. This can reduce inventory holding and shortage costs “because of a portfolio effect over the lead time from the supplier” (Eppen and Schrage 1981, p. 67).

#### The role of organization size

Large Chinese manufacturing companies have been shown to apply postponement more than small ones, as they may be able to afford it better (Huang and Li 2008). Similarly, participants from large hospitals consider postponement, component commonality, and transshipments more applicable with regard to examinations and treatments in order of decreasing strength of correlation and currently already apply product substitution in these services more than smaller hospitals.

### Managerial implications

Hospitals suffer from demand and lead time uncertainty especially for treatments and medications (RI7). Risk pooling can decrease these uncertainties and enable to offer the same service level at a lower cost and therefore improve the economic situation of hospitals (RI8).

This holistic research gives an overview of the methods that can achieve risk pooling and the status of their application (RI1) and applicability (RI2) to medications, capital goods, consumer goods, examinations, and treatments in different types of German hospitals (RI3 and RI4), as well as their interrelationships (RI6), restrictions (R9) and appraisal by administrators, physicians, and nurses (RI5). This allows managers to appraise risk pooling methods for their hospitals.

The empirical evidences described and analyzed in the present paper provide some hints for managers:

#### Choose suitable risk pooling methods and scope of application

Inventory pooling, transshipments, and product substitution for medications and consumer goods may be most applicable in hospitals (RI1 and RI2). Product substitution in treatments and examinations, however, may not be beneficial (RI8). Despite recommendations in the literature, form postponement may be unsuitable for hospitals due to the required efforts, delays in treatments, possibility of confusion, and liability issues (RI9).

Hospitals may focus on medications and consumer goods (RI1). Bottlenecks in the delivery of many drugs have been increasing for years due to manufacturers offshoring their production for cost reasons, fewer manufacturers producing an active ingredient and global supply chains concentrating on few manufacturing facilities, quality problems, delays in raw materials production and delivery, production setups, and market withdrawals (Müller 2019). The current COVID-19 pandemic has exacerbated supply bottlenecks for pharmaceuticals and created new ones for personal protective equipment and disinfectants (Müller 2020).

Demand pooling seems more suitable for medications, examinations and treatments, lead time pooling more for capital and consumer goods (RI7). Demand pooling and lead time pooling methods can be applied together to balance their disadvantages (RI6).

Risk pooling should only be applied to the uncertain portion of healthcare, as the plannable part may be provided at a lower cost without risk pooling (cf. Chopra and Meindl 2019).

Risk pooling should be used between parts of hospitals or hospitals that are exposed to different fluctuations in demand or lead times, since negative correlations between demands and lead times increase the overall reduction in variability and thus the advantages of risk pooling (Oeser 2015).

#### Consider differences in hospital type, hospital size, stakeholders, and restrictions

Hospitals of different sizes and public, non-profit and private hospitals differ, inter alia, in their objectives, market shares, outsourcing rate, labor productivity, profitability, ability to invest, nursing staff, participation in medical and emergency care (Augurzky et al. 2018) as well as the demand and lead time uncertainties they experience (RI7) and the application and applicability of risk pooling (RI3 and RI4). If risk pooling is to be implemented successfully, these differences, stakeholders, identified interrelationships, and restrictions need to be considered (RI3 − RI6, RI9).

This research shows that hospitals may benefit from risk pooling economically. For risk pooling to be beneficial, hospitals need to choose suitable risk pooling methods for their characteristics and uncertainties they face. In selecting risk pooling methods their advantages and disadvantages need to be quantified and weighed against each other. Hospitals also need to check if their uncertainties cannot be reduced more efficiently in other ways, such as by improving forecasting (Heil 2006), responsiveness, flexibility, or capability (Chopra and Sodhi 2004).

The administration and medical staff seem to have different opinions on the uncertainty of medication lead times and the applicability of centralized ordering, transshipments, and product substitution (RI5), which may not be fully explained by their belonging to different hospitals. Langabeer et al. (2009) already proposed that physicians and nurses may rather oppose efficiency projects. The variability of demand and lead times for different items could be objectively measured using the coefficient of variation and gain a common understanding of the real extent of uncertainties. Thus, the variability of different demands and lead times becomes comparable and those with the highest variabilities can be tackled first.

The application of risk pooling in healthcare requires willingness to exchange information and to cooperate, adequate IT infrastructure, compatibility, adherence to healthcare laws and regulations, and securing the immediate treatment of emergencies (RI9).

#### Reap the variability-reduction benefits of risk pooling

Hospitals may apply the risk pooling methods for other benefits such as centralized ordering for economies of scale or order splitting to decrease prices by having multiple suppliers compete. However, they may miss the risk pooling benefits these methods may also bring. Risk pooling can reduce variability in demand and / or lead time and thus enables to reduce costs (e.g. inventory and capacity costs) for a given level of healthcare service (e.g. product availability and treatment time), to increase the service level at a given cost, or a combination of both. However, this is not automatic (cf. Zepeda et al. 2014; Oeser and Romano 2016; Oeser 2019), but has to be tracked and implemented.

## Conclusions and further research

“One of the most powerful tools used to address variability in the supply chain is the concept of risk pooling” (Simchi-Levi et al. 2008, p. 48). This paper complements the large body of business logistics research on this topic by exploring how this concept is and can be applied in hospitals. Compared to other businesses, hospitals have extensive contact to their customers, the patients, whose recovery and life may also depend on the availability of medical supplies (Jacobs and Chase 2020).

Demands for medications, examinations and treatments and lead times for consumer and capital goods appear most uncertain. Applying risk pooling may reduce these uncertainties and the current strain on hospitals due to COVID-19 and improve the economic condition of hospitals. The administration and medical staff seem to differ in their assessments of the uncertainty and applicability of risk pooling methods, which should be discussed and resolved before implementing risk pooling.

German hospitals seem to use inventory pooling, transshipments, and product substitution the most and component commonality, order splitting, and postponement the least. Risk pooling is applied the most to medications and consumer goods and the least to treatments and examinations (services). These objects and services seem to be considered separately by the survey participants, but their interrelationships should be considered.

In addition to the current risk pooling usage, virtual pooling of capital goods, centralized ordering of consumer goods and order splitting of medications seem most applicable. The applicability of risk pooling methods may differ for large and small and public, non-profit and private hospitals.

Risk pooling in procurement (including common components) seems to be most strongly correlated with the application of risk pooling in storage. Hospitals may benefit from exploring risk pooling options further down the hospital supply chain.

Restrictions to applying risk pooling in hospitals include treating emergencies immediately, observing healthcare laws and regulations, and information exchange and cooperation.

As this study is based on data for Germany, studies in other countries may further enhance our knowledge on the application and applicability of risk pooling methods in healthcare.

The specific design, benefits and challenges of risk pooling methods should be analyzed in further detailed empirical studies in a wide variety of hospitals, for elective and non-elective cases, and inpatient and outpatient flows. As competition may be an issue, risk pooling implementation could first be tested within a hospital group under a common sponsorship. Afterwards, studies could investigate how hospitals may cooperate as part of a risk pooling concept without lifting competition between them in other areas.

For this explorative research a questionnaire survey was suitable. While survey research captures the respondents’ perceptions of object reality, this reality may be directly observed in further detailed field studies or experiments (Meredith et al. 1989).

Hospitals may lack in-house logistics expertise (Ageron et al. 2018) and rely on logistics service providers for delivering medical supplies, as supported by the free-text answers. Therefore, logistics service providers may play a critical role in implementing risk pooling in hospitals successfully. If they excel in understanding and fulfilling the needs of hospitals, they may also increase their revenues. This link between logistics outsourcing and risk pooling has not been analyzed yet.

Finally, this study may be repeated in the future to examine whether and how hospitals’ approach to risk pooling has changed because of the current COVID-19 pandemic that has increased demand and lead time uncertainties.

## Appendix

Online questionnaire.

1. Is your hospital public, non-profit or private? *Checkboxes*
2. Total number of employees: *Free text field*
3. Total number of beds: *Free text field*
4. Your position: administration, doctor, care (*checkboxes*); exact title (*free text field*)
5. How would you rate the economic situation in your hospital? *Six-point Likert scale (1: very good – 6: very bad)*
6. Which of the following objects/activities have uncertain or fluctuating demands?
  *Answer choices for 6 and 7: Medications, consumer goods, capital goods, examinations, treatments (checkboxes).*
  *Medications are drugs or other forms “of medicine that you take to prevent or to treat an illness” (*Oxford University Press 2020*). Consumer goods directly satisfy needs, such as food, surgical thread, cleaning materials, or prostheses (*Santos et al. 2018*). Medications are also consumer goods (*Santos et al. 2018*), but are considered separately due to their importance and often separate supply chain in hospitals (*Mc Guire 2015*;* Jacobs and Chase 2020*). Capital goods are used by the hospital for a longer period of time to produce healthcare services, such as autoclaves, incubators, electrocardiographs, furniture, or utensils (*Santos et al. 2018*). Examinations take “a close look at” patients “to see if there is anything wrong or to find the cause of a problem” (*Oxford University Press 2020*). Treatments are medical actions taken to alleviate or cure illnesses or injuries.*
7. Which of the following objects/activities have uncertain or fluctuating delivery/lead times?
8. Which of the following methods is applied or could be applied for the following objects/activities?
  - Consolidation/centralization of inventories of the following objects/for the following activities
    *Answer choices for a. − j.*
    *Checkboxes: medications (applied, applicable), consumer goods (applied, applicable), capital goods (applied, applicable), examinations (applied, applicable), treatments (applied, applicable). Comments/restrictions (free text field)*
  - Access to stocks / examinations or treatments of other own or third-party locations via information and communication technologies
  - Transport of stocks or transport of patients for examinations or treatments between locations (e.g. hospital parts or hospitals) in the case of shortages
  - Bundling of orders of different objects for different locations (e.g. departments or hospitals) or for different treatments or examinations
  - Distribution of orders of objects, examinations or treatments to several suppliers, deliveries or locations in order to compensate fluctuations in delivery, examination, or treatment times
  - Using identical components instead of individual components for several medications, consumer goods, capital goods, treatments or examinations
  - Postponing decisions in logistics, procurement, or service regarding the following objects and activities in order to be able to use up-to-date demand information.
    Examples: divisible tablets (medications), dilute generic disinfectant concentration with water instead of ordering different concentrations, cut tubes on rolls to the required size, fold compresses or cut to the required format (consumer goods), configure generic medical devices according to current requirements, combine modules as required (capital goods) (cf. Mc Guire 2015)
  - Sharing of capacities of different units or hospitals regarding the following objects or activities.
    Examples: Sharing storage capacities (for medications, consumer goods, capital goods), sharing examination capacities (pathology, laboratories) and treatment capacities (beds, surgery, nursing training, anesthesia, cardiology)
  - Combining different forms of objects/activities into one universal form (standardization) or reducing the number of variants.
    Examples: standardizing the administration form (tablets, capsules, syrup), strength (200 mg, 250 mg) or pack size of medications, standardizing needles, bandages, tubes, drainage, rubber or silicone catheter, gloves, X-ray film (consumer goods), standardizing devices (capital goods) (cf. Mc Guire 2015), standardizing examinations, standardizing treatments
  - Use of alternatives or substitutes, e. g. because the originally required drug, consumer good, capital good, examination or treatment is not available.

Thank you.
